# Characterizing the profiles of patients with acute concussion versus prolonged post-concussion symptoms in Ontario

**DOI:** 10.1038/s41598-023-44095-6

**Published:** 2023-10-20

**Authors:** Olivia F. T. Scott, Mikaela Bubna, Emily Boyko, Cindy Hunt, Vicki L. Kristman, Judith Gargaro, Mozhgan Khodadadi, Tharshini Chandra, Umme Saika Kabir, Shannon Kenrick-Rochon, Stephanie Cowle, Matthew J. Burke, Karl F. Zabjek, Anil Dosaj, Asma Mushtaque, Andrew J. Baker, Mark T. Bayley, CONNECT, Flora Matheson, Ruth Wilcock, Billie-Jo Hardie, Michael Cusimano, Shawn Marshall, Robin Green, Thomas Hoshizaki, James Hutchison, Tom Schweizier, Michael Hutchison, Justina Zych, David Murty, Maria Carmela Tartaglia

**Affiliations:** 1 Canadian Concussion Centre, University Health Network, Toronto, ON Canada; 2 Hamilton Health Sciences, Hamilton, ON Canada; 3 EPID@Work Research Institute, Lakehead University, Thunder Bay, ON Canada; 4 Head Injury Clinic, Department of Trauma and Neurosurgery, St. Michael’s Hospital, Toronto, ON Canada; 5 Dalla Lana School of Public Health, University of Toronto, Toronto, ON Canada; 6 Concussion Ontario Network: Neuroinformatics to Enhance Clinical Care and Translation, Toronto, ON Canada; 7 Department of Health Sciences, Lakehead University, Thunder Bay, ON Canada; 8 Neurotrauma Care Pathways Project, KITE Research Institute, University Health Network, Toronto, ON Canada; 9 Hull-Ellis Concussion Clinic, Toronto Rehabilitation Institute, University Health Network, Toronto, ON Canada; 10 Northern Ontario School of Medicine University, Thunder Bay, ON Canada; 11 Health Sciences North Research Institute, Sudbury, ON Canada; 12 Parachute, Toronto, ON Canada; 13 Neuropsychiatry Program, Division of Neurology, Department of Psychiatry, Department of Medicine, Sunnybrook Health Sciences Centre, University of Toronto, Toronto, ON Canada; 14 Hurvitz Brain Sciences Research Program, Sunnybrook Research Institute, Toronto, ON Canada; 15 Department of Physical Therapy, Temerty Faculty of Medicine, University of Toronto, Toronto, ON Canada; 16 KITE Research Institute, University Health Network, Toronto, ON Canada; 17 Brain Health and Wellness Research Program, St. Michael’s Hospital, Unity Health Toronto, Toronto, ON Canada; 18 Faculty of Medicine, Institute of Medical Science, University of Toronto, Toronto, ON Canada; 19 Department of Anesthesia, University of Toronto, Toronto, ON Canada; 20 Division of Physical Medicine and Rehabilitation, Temerty Medicine, University of Toronto, Toronto, ON Canada; 21 Division of Neurology, Toronto Western Hospital, University Health Network, Toronto, ON Canada; 22 Tanz Centre for Research in Neurodegenerative Diseases, University of Toronto, Toronto, ON Canada; 23 MAP Centre for Urban Health Solutions, St. Michael’s Hospital, Toronto, ON Canada; 24 Ontario Brain Injury Association, St. Catherines, ON Canada; 25 Li Ka Shing Knowledge Institute, St. Michael’s Hospital, Toronto, ON Canada; 26 Division of Neurosurgery, University of Toronto, Toronto, ON Canada; 27 Ottawa Hospital Research Institute, Ottawa, ON Canada; 28 Department of Medicine, University of Ottawa, Ottawa, ON Canada; 29 Toronto Rehabilitation Institute, University Health Network, Toronto, ON Canada; 30 Department of Rehabilitation Sciences, University of Toronto, Toronto, ON Canada; 31 School of Human Kinetics, University of Ottawa, Ottawa, ON Canada; 32 Department of Critical Care, The Hospital for Sick Children, Toronto, ON Canada; 33 Neuroscience and Mental Health Research Program, The Hospital for Sick Children Research Institute, Toronto, ON Canada; 34 Interdepartmental Division of Critical Care, University of Toronto, Toronto, ON Canada; 35 Department of Paediatrics, University of Toronto, Toronto, ON Canada; 36 Faculty of Medicine (Neurosurgery), University of Toronto, Toronto, ON Canada; 37 Neuroscience Research Program, St. Michael’s Hospital, Toronto, ON Canada; 38 Keenan Research Centre, St Michael’s Hospital, Toronto, ON Canada; 39 Faculty of Kinesiology and Physical Education, University of Toronto, Toronto, ON Canada; 40 MacIntosh Sport Medicine Clinic, University of Toronto, Toronto, ON Canada; 41 Trauma and Neurosurgery Program, St. Michael’s Hospital, Toronto, ON Canada

**Keywords:** Neuroscience, Neurology, Risk factors

## Abstract

Identifying vulnerability factors for developing persisting concussion symptoms is imperative for determining which patients may require specialized treatment. Using cross-sectional questionnaire data from an Ontario-wide observational concussion study, we compared patients with acute concussion (≤ 14 days) and prolonged post-concussion symptoms (PPCS) (≥ 90 days) on four factors of interest: sex, history of mental health disorders, history of headaches/migraines, and past concussions. Differences in profile between the two groups were also explored. 110 patients with acute concussion and 96 patients with PPCS were included in our study. The groups did not differ on the four factors of interest. Interestingly, both groups had greater proportions of females (acute concussion: 61.1% F; PPCS: 66.3% F). Patient profiles, however, differed wherein patients with PPCS were significantly older, more symptomatic, more likely to have been injured in a transportation-related incident, and more likely to live outside a Metropolitan city. These novel risk factors for persisting concussion symptoms require replication and highlight the need to re-evaluate previously identified risk factors as more and more concussions occur in non-athletes and different risk factors may be at play.

## Introduction

A concussion is a form of mild traumatic brain injury (mTBI) caused by a direct or indirect force to the head and may induce loss of consciousness, headaches, and personality changes^1^. In Ontario, over 1% of the population per year was diagnosed with a concussion from 2008 to 2016^2^. This number only accounts for patients who sought medical care, and thus, the true incidence may be greater^2^. While the majority of patients who suffer a concussion fully recover within days to weeks, approximately 10–20% experience persisting symptoms^3–6^. Persistent symptoms of concussion have no universally agreed-upon criteria, but recently, it was defined as a symptom lasting over 4 weeks for sports-related concussion in all age groups^7^. Typically, acute concussion is diagnosed up to 14 days after injury^8^. If symptoms persist beyond 3 months, patients are considered to have prolonged post-concussion symptoms (PPCS)^8–11^. In Ontario, the lasting effects of concussion symptoms result in an estimated additional healthcare cost of $110 million annually^12^. Identifying factors that influence the development of persistent symptoms is crucial to initiating targeted therapy early for patients at risk of slower recovery.

Existing literature comparing patients with acute concussion and PPCS is limited. Mahmood et al.^5^ found a higher proportion of females, adults aged 25 and older, and patients with more initial symptom burden in their persistent concussion group. Other studies reported that factors such as prior history of concussion(s), attention deficit hyperactivity disorder, and specific symptoms (e.g. difficulty concentrating, insomnia) increased the odds of being in a persistent versus acute symptom group^13,14^. However, these latter two studies were comprised of pediatric sports concussion patients and college athletes, and their persisting symptoms groups were defined as symptoms lasting ≥ 4 weeks rather than ≥ 90 days.

While numerous studies have attempted to identify factors contributing to the development of persistent symptoms of concussion^4,15–19^, systematic reviews highlight suboptimal methodologies in many of these studies^20–23^. Nonetheless, certain factors consistently emerge as important predictors. History of mental health disorders has been repeatedly associated with the development of persistent symptoms of concussion in large cohort studies^4,15,17,19^, and systematic reviews^21,22,24^. Recently, Langer et al.^4^ found that history of anxiety, depression, bipolar disorder, and personality disorders were all associated with a greater risk of persistent symptoms six months post injury.

A prior history of headaches/migraines has also been reported to influence the development of persistent symptoms. However Cnossen et al.^17^ found that pre-injury migraine or headache was only a significant independent predictor of symptom burden 6 months following mTBI, and not significant in a multivariable linear regression model. Langer et al.^4^ had similar findings, and a systematic review by Iverson et al. also demonstrated conflicting results, in turn suggesting that additional investigation is required^21^.

In addition to factors that influence the development of persistent symptoms, other factors may increase the risk of sustaining a concussion. There is increasing evidence that females are at a higher risk of sustaining a concussion from the same activity compared to males^25–27^. However, concussion studies often include more males^2,15,18,25,28^. This may be because more males participate in activities where brain injuries are common, such as contact sports, working in manufacturing or construction, and military personnel^25,29–31^. Female sex may also be a risk factor for developing persistent symptoms of concussion^5,15,32–34^. Thus, while more males sustain concussions, more females experience persisting symptoms. A history of previous concussions may be associated with sustaining a subsequent concussion^25,35^ and prolonged recovery^6,13,14,17,36,37^. Van Pelt et al.^25^ found that a history of previous concussions was associated with almost twice the risk of sustaining another concussion and Zemek et al.^36^ found that it increased the risk of persisting symptoms.

The primary aim of this study was to confirm whether patients with acute concussion versus PPCS significantly differed on four factors of interest: sex, history of mental health disorders, history of headaches/migraines, and history of past concussions. Given that only 10–20% of patients with concussion are reported to experience persisting symptoms^3–6^, one would predict a significant difference in prevalence of risk factors. We hypothesized that the PPCS group would have a significantly greater prevalence of history of mental health disorders and of headaches/migraines, and that there would be no differences between the groups for concussion history as it is a known risk factor for sustaining a concussion and for prolonged persisting symptoms of concussion. Additionally, we expected there to be significantly more males in the acute concussion group, and more females in the PPCS group. Our secondary aim was to explore differences in the demographic and injury-related profiles of patients with acute concussion versus PPCS to identify potential factors for inclusion in future longitudinal analyses.

## Methods

### Study design

This study used patient-reported data from participants enrolled in an observational multisite study called Concussion Ontario Network: Neuroinformatics to Enhance Clinical Care and Translation by Integrating Numbers Geographically (CONNECT-ing). CONNECT-ing is an ongoing study of physician-led concussion care sites and their multidisciplinary teams from across the province of Ontario. It involves the collection of a standardized set of common data elements (CDEs) from participants upon a visit to their respective concussion clinic, or shortly thereafter (within 2 weeks for most).

We report on aggregate data from patients attending one of seven CONNECT-ing clinical sites who completed the survey between November 2020 and January 2022. The sites represent different types of concussion clinics (2 sport, 1 acute care, 4 tertiary care), treat patients at different times since injury (acute, sub-acute, prolonged) and service patients across Ontario. All methods were carried out in accordance with relevant guidelines and regulations. The study was approved at all participating sites by the following Research Ethics Boards: Hamilton Integrated Research Ethics Board, Laurentian University Research Ethics Board, Unity Health Toronto Research Ethics Board, University Health Network Research Ethics Board, and Western University’s Health Sciences Ethics Board. All participants provided informed consent prior to enrollment.

### Participants

The inclusion criteria for the CONNECT-ing Study were (1) clinical diagnosis of a concussion consistent with the 2017 Berlin Consensus Statement^38^, (2) age ≥ 16 at time of consent and capable of consent, (3) able to complete the study questionnaires (with assistance if needed), and (4) Glasgow Coma Scale (GCS) ≥ 14. Patients across the entire spectrum of concussion were eligible (acute, sub-acute, prolonged). Patients were excluded if they had any abnormality on a Computed Tomography (CT) or routine Magnetic Resonance Imaging (MRI) scan, required a neurosurgical operative intervention, were intubated or admitted to an Intensive Care Unit, sustained multi system injuries requiring hospital admission, had a significant chronic neurological developmental delay resulting in communication difficulties, or if an alternate type of trauma was the primary event (i.e. if the concussion was instigated by a seizure, syncope, migraine, etc.).

For this analysis, only participants assigned to the acute concussion and PPCS groups based on time since injury were included. Acute concussion and PPCS were defined as ≤ 14 and ≥ 90 days between concussion and survey completion, respectively^8–11^. Participants in the subacute and prolonged symptom stages of concussion (15–29 and 30–89 days between concussion and survey completion, respectively) were excluded.

### Procedures and data management

Standardized and harmonized data collection procedures were implemented across sites. Participants completed the questionnaires either at their clinic visit or shortly thereafter (within 2 weeks for most). Participants reported their survey responses directly on the study database (REDCap) using a computer or portable tablet, or using pen and paper (in clinic or through mail). In the latter instance, the site Research Assistant (RA) subsequently entered the data into REDCap. Alternatively, where the questionnaires were part of the clinic’s standardized intake assessment, the site RA transferred this data to REDCap following patient consent. Embedded logic safeguards in REDCap ensured variables were entered within predetermined ranges. From REDCap, data were transferred to Brain-CODE for centralized storage. Brain-CODE is an open access, large-scale neuroinformatics platform that is developed and maintained by the Ontario Brain Institute (OBI)^39^.

### Study variables

The study questionnaires included questions recommended by several international scientific concussion agencies including the National Institute of Neurological Disorders and Stroke (NINDS)^40^, the Center for Disease Control (CDC)^41^, the Ohio State University Identification of TBI^42^, and the Ontario Guideline for Concussion/Mild Traumatic Brain Injury and Prolonged Symptoms, 3rd edition^37^. The questions on healthcare utilization have previously been used in concussion research^12^. This study reports on the following data collected from the CONNECT-ing Survey.

#### Demographics

Sex, age at time of last injury, marital/partner status, education, geographic region, ethnicity, annual household income, work status, job category.

#### Injury information

Mechanism of injury, days since injury.

#### Medical history

- Previous concussions
- Medical factors (e.g. cancer, thyroid disease)
- Vascular factors (e.g. diabetes, hypertension, high cholesterol)
- Pain (e.g. arthritis, fibromyalgia, back pain/injury)
- Developmental disorders (e.g. autism spectrum disorder, learning disabilities)
- Mental health disorders (e.g. depression, anxiety, post-traumatic stress disorder, schizophrenia, borderline personality disorder)
- Sleep disorders
- Vertigo/dizziness

#### Healthcare utilization

Number of visits to health services (Emergency Department [ED], family doctor, walk-in-clinic, rehab services, other) since the index concussion.

#### Symptoms-sport concussion assessment tool 5 (SCAT5)

The SCAT5 is a self-report symptom assessment tool that asks participants about 22 common concussion symptoms^43^. Symptom severity is rated on a 7-point Likert scale (0 = none, 1–2 = mild, 3–4 = moderate, 5–6 = severe). SCAT5 scores were subdivided by averaging the sum of symptom severity scores for four subcategories: somatic, cognitive, emotional, and fatigue, previously defined by Rădoi et al.^44^, except the variable confusion which was added to the cognitive subcategory. The somatic category included the following symptoms: headaches, pressure in head, neck pain, nausea or vomiting, dizziness, blurred vision balance problems, sensitivity to light, sensitivity to noise. The cognitive category included feeling slowed down, feeling like in a fog, don't feel right, difficulty concentrating, difficulty remembering, confusion. The emotional category included the following symptoms: more emotional, irritability, sadness, nervous or anxious. Finally, the fatigue/sleep category included fatigue or low energy, drowsiness, trouble falling asleep.

#### Perceived disability-Sheehan disability scale (SDS)

The SDS is a self-report scale that measures perceived disability^45^. Part of the assessment is a measurement of “days lost” and “days underproductive.” It asks participants to indicate how many days in the last week (0–7) did their symptoms cause them to miss school or work or leave them unable to carry out their normal daily responsibilities (days lost) and feel so impaired by their symptoms that even though they went to school or work or had other daily responsibilities, their productivity was reduced (days underproductive).

### Statistical analysis

The data were exported from OBI’s Brain-CODE REDCap database^39,46^, inclusive of entries from November 1, 2020, to January 31, 2022. Age at time of injury was calculated using the date of the index concussion and the month and year of each patient's date of birth, assuming all patients were born on the first day of the month. Mean patient age between patients with acute concussion versus PPCS was compared using a Mann–Whitney U test as the continuous data were of a non-normal distribution, as per a Shapiro–Wilk normality test. Supplementary SCAT5 data from the Toronto Western Hospital site were imported into the dataset to be included for analysis.

All analyses were conducted using IBM SPSS Statistics (version 28.0.1) and radar plots were created in Microsoft Excel (version 2108). Descriptive statistics and Chi-squared tests, as well as odds ratios and 95% confidence intervals for significant Chi-squared results were used to compare proportions of demographic and injury-related variables between the acute concussion and PPCS groups. Our primary aim was achieved by calculating odds ratios and 95% confidence intervals comparing PPCS and acute concussion on the primary factors of interest: sex, history of mental health disorders, history of headaches/migraines, and history of past concussions. For all tests, p values less than 0.05 were deemed statistically significant.

## Results

The cross-sectional dataset of 293 patients age ≥ 16 with concussion in Ontario contained 110 (37.5%) patients with acute concussion and 96 (32.8%) patients with PPCS. The average number of days since injury for the acute concussion and PPCS groups were 6 ± 3 and 717 ± 761, respectively. In the PPCS group, 43% were within 1 year, 23% were within 1–2 years and 14% were within 2–3 years. Only 20% were greater than 3 years since their concussion. Demographic and injury-related factors were contrasted between the two groups: sex, age, marital status, education, region, ethnicity, household income, current work status, job classification category, mechanism of injury, concussion history, and medical history variables (Table 1). Patient ages were significantly different between the two groups (U = 7630.5, p < 0.001), with the PPCS group being older in mean age (acute concussion: 32 ± 13; PPCS: 44 ± 16). When dividing patients in the acute concussion and PPCS groups into age categories of ≤ 39 and 40 + , an association between population and age was found (χ^2^ (_1, N=205_) = 34.64, p < 0.001) such that proportionately more patients with PPCS versus acute concussion were in the 40 + group (61.4% vs 21.1%, respectively). There were no significant differences with respect to sex distribution, acute concussion: 61.1% F; PPCS: 66.3% F (χ^2^_(1, N=203)_ = 0.591, p = 0.442). The average number of visits to health services since the index concussion was 5 ± 28 (acute concussion) and 68 ± 94 (PPCS). In terms of ED visits in particular, 88.2% of the acute concussion group and 62.5% of the PPCS group visited the ED at some point for their concussion (76.2% overall). The average number of days self-reported as underproductive in the week before survey completion for the acute concussion and PPCS groups were 3 ± 2.2 and 4 ± 2.7, respectively. Likewise, the average number of days reported as “lost” in the week before survey completion was 3 for both groups (acute concussion: ± 2.0; PPCS: ± 2.9).

**Table 1:** Demographics of patients age ≥ 16 with acute concussion and PPCS.

Variable	Categories	n (%)		p value^a^
		PPCS (n = 96), n (%)	Acute concussion	
			(n = 110), n (%)	
Sex	Female	63 (66.3)	66 (61.1)	0.442
	Male	32 (33.7)	42 (38.9)	
Age	≤ 29	22 (22.9)	56 (51.4)	< 0.001*
	30–39	15 (15.6)	30 (27.5)	
	40–49	24 (25.0)	12 (11.0)	
	50–59	20 (20.8)	7 (6.4)	
	60+	15 (15.6)	4 (3.7)	
Marital/partner status	Never married	34 (36.2)	71 (66.4)	< 0.001*
	Separated	6 (6.4)	4 (3.7)	
	Married/domestic partnership	44 (46.8)	29 (27.1)	
	Divorced	10 (10.6)	3 (2.8)	
Highest level of education completed	High school or less	6 (6.3)	21 (19.3)	0.034*
	Some college, no degree	18 (18.9)	23 (21.1)	
	Associate degree (academic, occupational, technical, or vocational programs)	12 (12.6)	4 (3.7)	
	Bachelor’s degree	33 (34.7)	34 (31.2)	
	Graduate or professional degree	25 (26.3)	26 (23.9)	
	Unknown	1 (1.1)	1 (0.9)	
Region (based on postal code)	Metropolitan Toronto	38 (41.3)	75 (75.0)	< 0.001*
	Central Ontario	25 (27.2)	12 (12.0)	
	Southwestern Ontario	14 (15.2)	6 (6.0)	
	Other	15 (16.3)	7 (7.0)	
Ethnicity	White	70 (74.5)	68 (63.6)	0.222
	Other	22 (23.4)	37 (34.6)	
	Prefer not to answer	2 (2.1)	2 (1.9)	
Annual household income	$49,999 or less	22 (23.2)	36 (33.3)	0.064
	$50,000–$99,999	21 (22.1)	31 (28.7)	
	$100,000–$199,999	24 (25.3)	17 (15.7)	
	$200,000 or more	12 (12.6)	4 (3.7)	
	Prefer not to answer	11 (11.6)	12 (11.1)	
	Do not know	5 (5.3)	8 (7.4)	
Current work status	Currently working	37 (38.9)	71 (65.1)	< 0.001*
	Disabled	29 (30.5)	5 (4.6)	
	Temporary layoff, sick, or other leave	8 (8.4)	2 (1.8)	
	Student or retired	16 (16.8)	26 (23.9)	
	Other	5 (5.3)	5 (4.6)	
Job classification category	None	18 (19.4)	29 (26.9)	0.252
	Craft worker	2 (2.2)	3 (2.8)	
	Official/manager	8 (8.6)	16 (14.8)	
	Operative	2 (2.2)	3 (2.8)	
	Professional	40 (43.0)	36 (33.3)	
	Laborer/helper	1 (1.1)	1 (0.9)	
	Technician	0 (0.0)	2 (1.9)	
	Service worker	7 (7.5)	2 (1.9)	
	Sales worker	3 (3.2)	6 (5.6)	
	Administrative support worker	7 (7.5)	3 (2.8)	
	Unknown	5 (5.4)	7 (6.5)	
Mechanism of injury	Falls/flying and falling objects	26 (27.1)	40 (36.4)	< 0.001*
	Transportation-related	38 (39.6)	13 (11.8)	
	Violence-related	4 (4.2)	4 (3.6)	
	Sport/exercise-related	13 (13.5)	26 (23.6)	
	Other	15 (15.6)	27 (24.5)	
Previous diagnosis of concussion(s)	Yes	42 (44.7)	39 (36.1)	0.215
	No	52 (55.3)	69 (63.9)	
Medical history: medical factors^b^	Yes	26 (27.4)	24 (21.8)	0.356
	No	69 (72.6)	86 (78.2)	
Medical history: vascular factors^c^	Yes	1 (1.1)	1 (0.9)	0.917
	No	94 (98.9)	109 (99.1)	
Medical history: pain factors^d^	Yes	2 (2.1)	11 (10.0)	0.021*
	No	93 (97.9)	99 (90.0)	
Medical history: developmental disorders^e^	Yes	8 (8.4)	15 (13.6)	0.238
	No	87 (91.6)	95 (86.4)	
Medical history: sleep disorder	Yes	9 (9.5)	8 (7.3)	0.569
	No	86 (90.5)	102 (92.7)	
Medical history: vertigo/dizziness	Yes	9 (9.5)	3 (2.7)	0.040*
	No	86 (90.5)	107 (97.3)	

*PPCS* prolonged post-concussion symptoms.

*Significant p values (< 0.05).

^a^Values are from Chi-squared tests.

^b^Medical factors e.g. cancer, thyroid disease.

^c^Vascular factors e.g. diabetes, hypertension, high cholesterol.

^d^Pain factors e.g. arthritis, fibromyalgia, back pain/injury.

^e^Developmental factors e.g. autism spectrum disorder, learning disabilities.

Demographic variables including age, mechanism of injury, and region yielded significant Chi-squared p values indicating an association between these variables and concussion status (Table 1). They were also of interest for this exploratory analysis as they are possible targets for concussion prevention, therefore odds ratios were calculated for these variables.

The youngest age category (≤ 29) was used as the referent for age odds ratios. There were three significant odds ratios between the referent and age categories: 40–49 [odds ratio (OR) = 5.09, 95% CI 2.17–11.92], 50–59 (OR = 7.27, 95% CI 2.70–19.60), and 60+ (OR = 9.55, 95% CI 2.85–31.97) (Table 2). Thus, the likelihood that an individual would be in an older age group (40–49, 50–59, 60+) was higher for patients with PPCS versus acute concussion when compared to those 29 or younger.

**Table 2:** Odds ratios for age, region, and mechanism of injury variables among patients age ≥ 16 with acute concussion and PPCS.

Variable	PPCS (n = 96), n (%)	Acute concussion (n = 110), n (%)	OR	95% CI
Age				
< 29	22 (22.9)	56 (51.4)	1.00	–
30–39	15 (15.6)	30 (27.5)	1.27	0.58–2.80
40–49	24 (25.0)	12 (11.0)	5.09	2.17–11.92
50–59	20 (20.8)	7 (6.4)	7.27	2.70–19.60
60+	15 (15.6)	4 (3.7)	9.55	2.85–31.97
Region				
Metropolitan Toronto	38 (41.3)	75 (75.0)	1.00	–
Central Ontario	25 (27.2)	12 (12.0)	4.11	1.86–9.07
Southwestern Ontario	14 (15.2)	6 (6.0)	4.61	1.64–12.95
Other	15 (16.3)	7 (7.0)	4.23	1.59–11.25
Mechanism of injury				
Falls/flying and falling objects	26 (27.1)	40 (36.4)	1.00	–
Transportation-related	38 (39.6)	13 (11.8)	4.50	2.02–10.02
Violence-related	4 (4.2)	4 (3.6)	1.54	0.35–6.71
Sport/exercise-related	13 (13.5)	26 (23.6)	0.77	0.34–1.76
Other	15 (15.6)	27 (24.5)	0.85	0.38–1.89

*PPCS* prolonged post-concussion symptoms, *OR* odds ratio, *CI* confidence interval.

Metropolitan Toronto was used as the referent category for region odds ratios. Significant odds ratios were found between Metropolitan Toronto and all the remaining regions: Central Ontario (OR = 4.11, 95% CI 1.86–9.07), Southwestern Ontario (OR = 4.61, 95% CI 1.64–12.95), and Other (OR = 4.23, 95% CI 1.59–11.25) (Table 2). The odds of living in Central Ontario, Southwestern Ontario, or elsewhere were greater for patients with PPCS than acute concussion, when compared to those living in Metropolitan Toronto.

Falls/flying and falling objects was used as the referent category for mechanism of injury odds ratios. A significant odds ratio was found between falls/flying and falling objects and transportation-related injuries (OR = 4.50; 95% CI 2.02–10.02) indicating that transportation-related injuries were 4.5 times higher in the PPCS group than in the acute concussion group, when compared to those whose concussion was caused by falls/flying and falling objects (Table 2).

**Figure 1:**
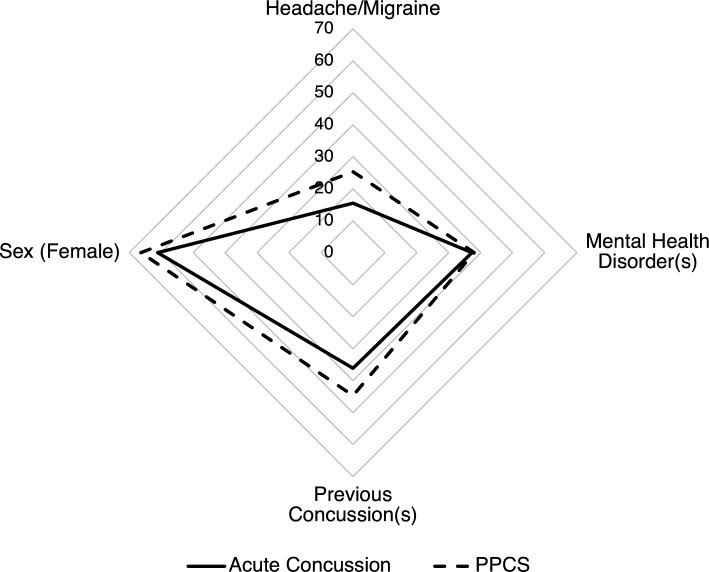
Radar plot for profiles of patients age ≥ 16 with acute concussion and PPCS based on variables of interest (Table 3). Scale is the percentage of patients who are female, or who answered yes to having a history of headache/migraine, a history of mental health disorders, or previous concussions.

The four primary factors of interest were not significantly different between the acute concussion and PPCS groups (sex, history of mental health disorders, history of headaches/migraines, concussion history) (Table 3). Proportions between patients with acute concussion and PPCS for these factors of interest were used to create profiles for patients with acute concussion and PPCS (Fig. 1).

**Table 3:** Categorical variables of interest for patients age ≥ 16 with acute concussion and PPCS.

Variable	PPCS (n = 96), n (%)	Acute concussion (n = 110), n (%)	OR	95% CI
Sex				
Female	63 (66.3)	66 (61.1)	1.25	0.70–2.22
Male	32 (33.7)	42 (38.9)		
History of mental health disorders^a^				
Yes	36 (37.9)	41 (37.3)	1.03	0.58–1.82
No	59 (62.1)	69 (62.7)		
History of headaches/migraines				
Yes	24 (25.3)	17 (15.5)	1.85	0.92–3.70
No	71 (74.7)	93 (84.5)		
Number of previous concussions				
0	52 (55.3)	69 (63.9)	1.00	–
1–2	24 (25.6)	23 (21.3)	1.38	0.70–2.71
≥ 3	18 (19.1)	16 (14.8)	1.49	0.69–3.20

*PPCS* prolonged post-concussion symptoms, *OR* odds ratio, *CI* confidence interval.

^a^Mental health disorders e.g. depression, anxiety, post-traumatic stress disorder, schizophrenia, borderline personality disorder.

**Figure 2:**
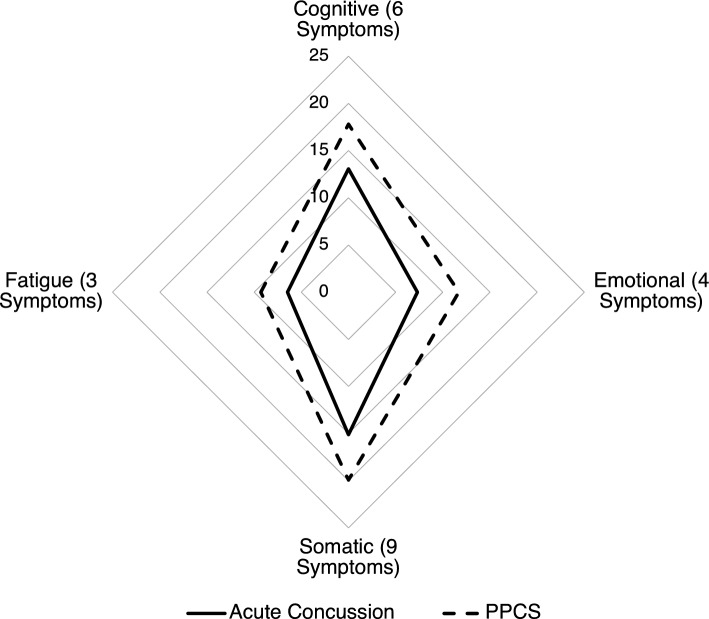
Radar plot of mean summed SCAT5 symptom scores for variables subcategorized by cognitive, emotional, somatic, and fatigue/sleep comparing patients age  ≥ 16 with acute concussion (n = 99) and with PPCS (n = 43).

Averaged subcategorized SCAT5 scores were compared between patients with acute concussion and PPCS; subcategories were cognitive, emotional, somatic, and fatigue (Fig. 2). Patients (n = 64) were excluded due to incomplete or missing SCAT5 data, as not all participating sites administered the SCAT5.

## Discussion

The primary aim of this study was to evaluate whether patients with acute concussion and PPCS differed on four factors of interest: sex, history of mental health disorders, past concussions, and history of headaches/migraines. Given that only 10–20% of acute concussions are reported to evolve into persisting symptoms^3–6^, we predicted significant differences in prevalence of risk factors. Contrary to our hypothesis, no significant differences were found between the two groups for history of mental health disorders, headache/migraine history, and sex. However, trends were observed in the expected direction. As expected, we found no significant differences between the groups for concussion history as it is both a risk factor for sustaining a concussion and for developing persistent symptoms. The most unexpected finding was a higher proportion of females than males in both groups.

The secondary aim was to explore differences in injury-related and demographic factors between the acute concussion and PPCS groups. The PPCS group had higher symptom scores in all four domains (Fig. 2). They were also significantly older, more likely to be injured in transportation-related incidents, and more likely to live outside of Metropolitan Toronto.

### Sex

Regarding sex as a risk factor for sustaining a concussion, we expected to find significantly more males in the acute concussion group due to greater exposure to environments where concussions are common (high-risk labour, contact sports) and because most concussion studies have more males than females^2,15,18,25,28^. However, the majority of our acute concussion group was female (61.1%). This may be partially explained by the timing of recruitment during the COVID-19 pandemic wherein fewer people were participating in high-contact sports, and high-risk workplaces such as construction sites were shut down. This may have created a more equal opportunity for concussion among males and females, and thus overall more concussions among females due to their elevated risk of concussion compared to males for the same activity^25–27^. Indeed, others have also highlighted that in the recent past, more females than males are suffering from concussions in sex-comparable sports^27,47^. Thus, as more females take part in activities that were historically dominated by males (and where concussions are common) we may observe a changing trend towards more concussions in females than males.

As expected there were more females in the PPCS group, since female sex is a risk factor for persisting symptoms of concussion^5,15,32–34^. Greater tendencies among females to seek care and report symptoms for medical conditions may have influenced these results^48,49^. The greater proportion of females in our sample compared to other studies may have also affected the results of other factors such as history of mental health disorders and headaches/migraines where sex may be a mediating factor. Thus, instead of correcting for sex as a confounder, it may be more informative for future studies to disaggregate the data by sex to further understand its effect on outcome.

### Previous concussions

As expected, since previous concussions can be a risk factor for prolonging concussion recovery^6,13,14,17,36,37^ and for sustaining a subsequent concussion^25,35^ we found no significant differences between the acute concussion and PPCS groups for this factor. In systematic reviews, Iverson et al.^21^ and Silverberg et al.^22^ found that a history of prior concussions was an important predictor of poor recovery or mTBI outcome in approximately 50% and 17% of their included studies that assessed this factor, respectively. Van Pelt et al.^25^ found it doubled the risk of sustaining another concussion. The number of previous concussions and the time between previous concussions and the presenting concussion may be modifying factors^22^, as well as whether the patient had recovered from their prior concussion. Additionally, distinguishing between prior mTBIs/concussions versus more severe TBIs should be considered since some studies do not make this distinction^4,17^.

### Mental health

History of mental health disorders has consistently been reported as a risk factor for persistent symptoms of concussion^4,15,17,19^. In contrast, we found no difference in mental health disorder history between patients with acute concussion (37.3%) and PPCS (37.9%). Differences in study sample may explain this finding. Many studies that report history of mental health disorders as a risk factor for persistent symptoms recruit patients from the ED and follow their recovery over time^15,17,19^ while we directly compared patients with acute concussion versus PPCS. Additionally, the greater proportion of females in both the acute concussion and PPCS groups in our study and the evidence that mental health disorders are more prevalent among females in the general population^50–54^ may have influenced our results, such that with similar proportions of females in both the acute concussion and PPCS groups, the effect of mental health was muted. Sex, which may itself be a risk factor for developing persistent symptoms, may therefore be one of the factors that mediates the role of mental health in the development of persistent symptoms. Studies should continue to assess this factor. Notably, another study also found that history of mental health disorders was not an important predictor^18^.

### Headaches/migraines

Prior research suggests that a history of headaches/migraines is associated with a longer recovery from concussion^36,55^, however not all studies have found this^21^. We did not find any significant differences between the two groups for this factor. Again, the fact that our acute concussion and PPCS samples did not significantly differ with respect to sex may have influenced our results given that headaches/migraines are more common in females^51,56–59^.

### Symptoms

The SCAT5 was used to visually compare perceived symptom severity between the acute concussion and PPCS groups over four symptom domains: fatigue, emotional, cognitive, and somatic. The resulting Radar plot (Fig. 2) suggests that the PPCS group had a higher perceived symptom severity than the acute concussion group in all four domains. We found that the PPCS group had a higher prevalence of fatigue symptoms. It is therefore not surprising that we also found that the PPCS group scored higher than the acute concussion group in the emotional symptoms domain as fatigue/sleep disturbances and emotional symptoms are correlated in concussion^60^. This is consistent with a longitudinal study that found that patients had an increase in emotional symptoms at 2–3 months compared to within 14 days of their concussion^32^.

With respect to cognitive symptoms, studies show that for patients with acute concussion, cognitive impairment improves within 3–7 days following concussion^61,62^. In patients with persisting symptoms, some studies have shown that cognitive symptoms can persist for years after a concussion^63–65^. Our results align with these studies.

We also found that somatic symptom scores were higher in the PPCS group versus the acute concussion group. This is consistent with research showing that reporting a high number of somatic symptoms in the acute phase is the best predictor of more PPCSs’ and longer lasting symptoms^66–68^. Importantly, our analysis on symptoms was limited to a smaller sample size as not all sites administered the SCAT5. Further studies examining symptomology and any potentially confounding factors are needed as there remain gaps about symptom profiles that may be more predictive of chronicity.

### Mechanism of injury

Regarding mechanism of injury, transportation-related injuries (e.g. motor vehicle, bicycle, and motorcycle incidents) were significantly more common in the PPCS group than the acute concussion group. This suggests that sustaining a concussion in a transportation-related incident may increase the risk of developing persistent symptoms. Tarkenton et al.^69^ found that youth who sustained a concussion in a motor vehicle collision (MVC) reported significantly higher persistent symptom severity and frequency compared to participants who had a sport-related concussion, and Varriano et al.^70^ found that after a single concussion, persistent symptoms were most common when the mechanism of injury was an MVC. It has been suggested that additional psychological stress associated with MVCs, the overlap between post-traumatic stress symptoms and persistent symptoms of concussion, and potentially greater forces on the brain in MVCs may explain why they appear to increase the risk of persistent symptoms^69,71^. It has also been suggested that involvement in litigation, which is most common in patients who sustained their concussion in an MVC^72^, is associated with persistent symptoms^73,74^.

### Age

The average age of patients in the PPCS group was significantly greater than that of patients in the acute concussion group (44 ± 16 versus 32 ± 13, respectively), suggesting that middle and older age may be a risk factor for developing persistent symptoms of concussion. This finding is consistent with other studies^4,19,75^. What remains less clear is the approximate age at which recovery begins to be negatively affected. While Langer et al.^4^ found that age 61+ was associated with worse recovery, King^75^ found that persistent symptoms were most common in patients aged 40+, and van der Naalt et al.^19^ found that recovery at 6 months was worst for the 40–64 age group when compared to the < 40 and 65+ groups. The results from our analysis comparing patients with acute concussion and PPCS age ≤ 39 versus 40+ are most consistent with the latter two studies such that we found an association between population and age, with proportionately more patients with PPCS in the 40+ group. When broken down further, we found significantly more patients in the PPCS group than in the acute concussion group for the older (40–49, 50–59, and 60+) age groups when compared to those 29 or younger. It has been postulated that decreased cognitive reserve associated with increased age may contribute to slower recovery following concussion in patients who are older^4,76^.

Nonetheless, not all studies have found that older age is associated with poor outcome^15,18,77,78^, and the need for additional investigation has been recognized^17^. Similar to sex, perhaps age is a mediating factor in the development of persistent symptoms. Indeed, van der Naalt et al.^19^ found that age interacted with education, and Booker et al.^15^ suggested that patients who are older may experience a smaller change in relative function if they have pre-injury reduced functionality. The extensive demographic and injury-related variables collected in the CONNECT-ing study will allow us to further elucidate the role of age in the development of persistent symptoms of concussion.

### Confounded by chronicity

While some variables that are more prominent in the PPCS group may suggest importance in the development of persistent symptoms, others may be confounded by chronicity. For example, differences between the acute concussion and PPCS groups for average number of healthcare visits and work status may be a factor of time. While we did find that more patients in the PPCS group were not working, it was surprising that so few patients in the acute concussion group were temporarily off work in the two weeks immediately following their concussion.

With respect to the SDS’s “days lost” and “days underproductive” questions, several factors may have influenced our results (similar average scores for both groups). For patients who completed the questionnaire just three days after their concussion, the maximum number of days lost and days underproductive due to the concussion would be three. For the PPCS group, some patients may have learned to manage their symptoms over time, therefore also potentially reporting 3 days lost/underproductive rather than more. Analyzing reporting patterns and potential confounders may help clarify the use and interpretation of the SDS for patients with concussion.

### Other demographics

Our study investigated other factors such as medical history, marital status and some social determinants of health. With respect to medical history only pain and dizziness/vertigo were different between the acute concussion and PPCS groups with more pain in the acute concussion group and more vertigo/dizziness in the PPCS group. The other medical factors (e.g. vascular factors, developmental disorders, sleep disorders) were not different between the two groups (Table 1). With respect to marital status, the PPCS group was more likely to be married, possibly due to this group being older. In terms of some social determinants of health we assessed, for education, a greater proportion of patients in the acute concussion group completed high school or less, possibly due to this group being younger. Furthermore, we did not find differences for ethnicity, yearly income, or job classification. Some of these variables may require a larger sample size to investigate their role.

One demographic factor that was significantly different between the two groups was our geographic data. Overall, we found that patients in the PPCS group were more likely to be from outside the Greater Toronto Area (GTA). Perhaps the dense population of the GTA leads to barriers when accessing care for persisting symptoms due to long wait times. Indeed, a study from Toronto, Canada found that there was an average waiting time of 10 months for tertiary care following an mTBI^12^.

Capturing patients with concussion across the province of Ontario is a unique strength of CONNECT-ing. While other studies focus on more confined populations such as only patients accessing healthcare in Toronto^12,79^ or other urban centres, this study included patients from Northern, Eastern, Southwestern, and Central Ontario, as well as Metropolitan Toronto, thus increasing generalizability. While some categories (Eastern and Northern Ontario) had to be collapsed due to small numbers, and while the proportions of patients from each region does not exactly mirror that of Ontario Census data (see Supplementary Table S1 online)^80^, we were nonetheless successful in recruiting patients from across the province. Studies targeted at how geographical location and urban versus rural settings contribute to concussion care and recovery may reveal important information to guide resource allocation.

A critical component for accelerating concussion research has been the call by international scientific experts to endorse the use of CDEs^81^. Using CDEs for concussion facilitates data sharing and harmonization from multiple sites to support the capacity to compare or combine data from different clinical environments in meaningful ways^82^. Currently in Ontario, CDEs are not widely used in concussion clinics and research, making it difficult to study concussion from wider clinical practice and geographic perspectives. The multi-site CONNECT-ing study addresses this gap by implementing standardized measures for research, with the overall goal of improving clinical care.

### Potential reasons for lack of differences between the acute concussion and PPCS groups

In addition to the potential role of sex in influencing the lack of significant findings for some previously identified risk factors, the prevalence of patients who develop persisting symptoms may have also influenced our results. While many studies suggest that only 10–20% of patients with concussion experience persisting symptoms^3–6^, there is increasing evidence that this is an underestimate^83^. If the true prevalence is closer to 50% as McInnes et al.^83^ suggest, we would indeed expect patients with acute concussion to be more similar to patients with PPCS for these risk factors.

### Limitations

We acknowledge that our study has some limitations. This is a cross-sectional study and a longitudinal study with follow-up data on all participants would be more accurate in terms of identifying factors that promote versus prevent the development of persistent symptoms. It would also reduce recall bias which likely affected our PPCS group. A second limitation is the sample size and use of categorical data. Our relatively small sample size provided less statistical power, and thus we may not have detected all the differences between the two groups. It also limited the focus of our study to four main variables of interest and inflated our odds ratios. Additionally, the use of categorical data limited the types of analyses we could conduct and we did not have enough participants to stratify the results by sex or conduct any multivariable analyses. The lack of standardized criteria for PPCS may be a limitation to all studies like ours. Our cut-off of ≥ 90 days was based on previous data, however there are no biomarkers to support this^8–11^. Furthermore, our sample did not adequately capture the older adult population. Indeed, older adults tend to be less represented in concussion research^19,84^. It is possible that older adults are not being referred to concussion clinics or may present to hospitals/clinics with more severe concurrent injuries, resulting in less focus on the concussion and potentially also excluding them from CONNECT-ing. Also important to note is that our population was ≥ 16 years of age and so our results may not apply to children.

Importantly, this study reports on data collected during the COVID-19 pandemic. The closure of many activities associated with concussion (e.g. high contact sports and certain work sites) and the decreased number of drivers on the road during this time may have affected the demographics of our sample and observed proportions of mechanism of injury. The present study also did not assess litigation which may have influenced our results. With respect to demographics, it may have decreased concussion incidence among males, as described above. The pandemic may have also affected some patients’ access to medical care, possibly also affecting the demographics of our sample.

Lastly, CONNECT-ing only captures patients who seek care for their concussion. Thus, patients who recover soon after their concussion or do not access any medical care are not captured. This may have also made the acute concussion group more similar to the PPCS group, contributing to the lack of differences observed. Similarly, since some patients in the acute concussion group would be expected to eventually meet the criteria for PPCS (approximately 10–20%), this creates some overlap between the two groups, especially with respect to demographics and pre-injury variables. Thus, this may have also contributed to the lack of differences observed.

## Conclusion

In order to identify patients requiring specialized interventions early on after concussion, it is critical to determine factors contributing to sustaining a concussion and to the development of persistent symptoms. This is one of few studies to concurrently assess patients with acute concussion and PPCS. While some studies evaluate patients with concussion only in the acute concussion phase^79,85^ or only patients with PPCS^18,86^, the direct comparison of the two is less prevalent and may provide a unique lens to this area of research. In this study, we found that the acute concussion and PPCS groups did not significantly differ on some previously identified risk factors for persistent symptoms of concussion: history of mental health disorders, history of headaches/migraines, and female sex. This is surprising given that previous literature stresses that only 10–20% of patients with concussion develop persisting symptoms^3–6^. There is however increasing evidence that this may be an underestimate^83^. As expected, the acute concussion and PPCS groups also did not differ with respect to prior concussions, as this is a risk factor for sustaining a concussion and for persistent symptoms. We did, however, identify some differences between the two groups with respect to age, mechanism of injury, and geographical region. The lack of sex differences that we observed between the groups suggests that sex may be one of the factors that mediates the effects of some previously identified risk factors for concussion. This requires further investigation. The CONNECT-ing dataset, which reflects a clinical cohort, offers a unique opportunity to explore concussion in a real-world setting that may reflect changing trends in concussion.

### Supplementary Information

Supplementary Information.

## Data Availability

The original, de-identified data (including study protocol and data dictionaries) will be available through Brain-CODE (https://www.braincode.ca). Requests to access these datasets should be directed to info@braininstitute.ca.
