# Author Correction: Characterizing the profiles of patients with acute concussion versus prolonged post-concussion symptoms in Ontario

**DOI:** 10.1038/s41598-024-55261-9

**Published:** 2024-02-27

**Authors:** Olivia F. T. Scott, Mikaela Bubna, Emily Boyko, Cindy Hunt, Vicki L. Kristman, Judith Gargaro, Mozhgan Khodadadi, Tharshini Chandra, Umme Saika Kabir, Shannon Kenrick-Rochon, Stephanie Cowle, Matthew J. Burke, Karl F. Zabjek, Anil Dosaj, Asma Mushtaque, Andrew J. Baker, Mark T. Bayley, CONNECT, Flora Matheson, Ruth Wilcock, Billie-Jo Hardie, Michael Cusimano, Shawn Marshall, Robin Green, Thomas Hoshizaki, James Hutchison, Tom Schweizier, Michael Hutchison, Justina Zych, David Murty, Maria Carmela Tartaglia

**Affiliations:** 1 Canadian Concussion Centre, University Health Network, Toronto, ON Canada; 2 Hamilton Health Sciences, Hamilton, ON Canada; 3 EPID@Work Research Institute, Lakehead University, Thunder Bay, ON Canada; 4 Head Injury Clinic, Department of Trauma and Neurosurgery, St. Michael’s Hospital, Toronto, ON Canada; 5 Dalla Lana School of Public Health, University of Toronto, Toronto, ON Canada; 6 Concussion Ontario Network: Neuroinformatics to Enhance Clinical Care and Translation, Toronto, ON Canada; 7 Department of Health Sciences, Lakehead University, Thunder Bay, ON Canada; 8 Neurotrauma Care Pathways Project, KITE Research Institute, University Health Network, Toronto, ON Canada; 9 Hull-Ellis Concussion Clinic, Toronto Rehabilitation Institute, University Health Network, Toronto, ON Canada; 10 Northern Ontario School of Medicine University, Thunder Bay, ON Canada; 11 Health Sciences North Research Institute, Sudbury, ON Canada; 12 Parachute, Toronto, ON Canada; 13 Neuropsychiatry Program, Division of Neurology, Department of Psychiatry, Department of Medicine, Sunnybrook Health Sciences Centre, University of Toronto, Toronto, ON Canada; 14 Hurvitz Brain Sciences Research Program, Sunnybrook Research Institute, Toronto, ON Canada; 15 Department of Physical Therapy, Temerty Faculty of Medicine, University of Toronto, Toronto, ON Canada; 16 KITE Research Institute, University Health Network, Toronto, ON Canada; 17 Brain Health and Wellness Research Program, St. Michael’s Hospital, Unity Health Toronto, Toronto, ON Canada; 18 Faculty of Medicine, Institute of Medical Science, University of Toronto, Toronto, ON Canada; 19 Department of Anesthesia, University of Toronto, Toronto, ON Canada; 20 Division of Physical Medicine and Rehabilitation, Temerty Medicine, University of Toronto, Toronto, ON Canada; 21 Division of Neurology, Toronto Western Hospital, University Health Network, Toronto, ON Canada; 22 Tanz Centre for Research in Neurodegenerative Diseases, University of Toronto, Toronto, ON Canada; 23 MAP Centre for Urban Health Solutions, St. Michael’s Hospital, Toronto, ON Canada; 24 Ontario Brain Injury Association, St. Catherines, ON Canada; 25 Li Ka Shing Knowledge Institute, St. Michael’s Hospital, Toronto, ON Canada; 26 Division of Neurosurgery, University of Toronto, Toronto, ON Canada; 27 Ottawa Hospital Research Institute, Ottawa, ON Canada; 28 Department of Medicine, University of Ottawa, Ottawa, ON Canada; 29 Toronto Rehabilitation Institute, University Health Network, Toronto, ON Canada; 30 Department of Rehabilitation Sciences, University of Toronto, Toronto, ON Canada; 31 School of Human Kinetics, University of Ottawa, Ottawa, ON Canada; 32 Department of Critical Care, The Hospital for Sick Children, Toronto, ON Canada; 33 Neuroscience and Mental Health Research Program, The Hospital for Sick Children Research Institute, Toronto, ON Canada; 34 Interdepartmental Division of Critical Care, University of Toronto, Toronto, ON Canada; 35 Department of Paediatrics, University of Toronto, Toronto, ON Canada; 36 Faculty of Medicine (Neurosurgery), University of Toronto, Toronto, ON Canada; 37 Neuroscience Research Program, St. Michael’s Hospital, Toronto, ON Canada; 38 Keenan Research Centre, St Michael’s Hospital, Toronto, ON Canada; 39 Faculty of Kinesiology and Physical Education, University of Toronto, Toronto, ON Canada; 40 MacIntosh Sport Medicine Clinic, University of Toronto, Toronto, ON Canada; 41 Trauma and Neurosurgery Program, St. Michael’s Hospital, Toronto, ON Canada

Correction to: *Scientific Reports* 10.1038/s41598-023-44095-6, published online 20 October 2023

The Data availability section in the original version of this Article was incorrect.

“Data in a de-identified format will be made available upon reasonable request to the corresponding author.”

now reads:

“The original, de-identified data (including study protocol and data dictionaries) will be available through Brain-CODE (www.braincode.ca). Requests to access these datasets should be directed to info@braininstitute.ca.”

The original Article has been corrected.

