# Supplementary material for: Characterizing the profiles of patients with acute concussion versus prolonged post-concussion symptoms in Ontario
Source: Sci Rep. 2023 Oct 20;13:17955. doi: 10.1038/s41598-023-44095-6 (PMC10589269; doi:10.1038/s41598-023-44095-6)
Supplement: Supplementary file 1 — Supplementary Information. [file 41598_2023_44095_MOESM1_ESM.pdf]

Supplementary Data

Characterizing the profiles of patients with acute concussion versus prolonged post-concussion symptoms in Ontario

Olivia FT Scott, Mikaela Bubna, Emily Boyko, Cindy Hunt, Vicki L Kristman, Judith Gargaro, Mozhgan Khodadadi, Tharshini Chandra, Umme Saika Kabir, Shannon Kenrick-Rochon, Stephanie Cowle, Matthew J Burke, Karl F Zabjek, Anil Dosaj, Asma Mushtaque, Andrew J Baker, Mark T Bayley, CONNECT, and Maria Carmela Tartaglia

Supplementary Table S1 2016 Ontario Census Data for Geographical Location versus CONNECT-ing Geographical Distribution[1]. n=110 acute concussion; n=96 PPCS

	2016 Census n (%)	Acute Concussion n (%)	PPCS n (%)	Acute Concussion + PPCS n (%)
**Eastern Ontario**	2,050,397 (15.2)	3 (3.0)	2 (2.2)	5 (2.4)
**Central Ontario**	5,243,100 (39.0)	13 (12.0)	26 (27.2)	39 (18.9)
**Metropolitan Toronto**	2,732,139 (20.3)	83 (75.0)	40 (41.3)	123 (59.7)
**Southwestern Ontario**	2,583,859 (19.2)	7 (6.0)	15 (15.2)	22 (10.7)
**Northern Ontario**	838,997 (6.2)	2 (2.0)	8 (8.7)	10 (4.9)
**Other**	NA	2 (2.0)	5 (5.4)	7 (3.4)
**Total**	13,448,492 (100)	110 (100)	96 (100)	206 (100)

PPCS, prolonged post-concussion symptoms

References

1. Statistics Canada. *Population and Dwelling Count Highlight Tables, 2016 Census*. (2018).
